# F61. THE RELATIONSHIP OF AGE AND SYMPTOMS WITH COGNITIVE PLANNING IN SCHIZOPHRENIA

**DOI:** 10.1093/schbul/sby017.592

**Published:** 2018-04-01

**Authors:** Dimitrios Kontis, Alexandra Giannakopoulou, Eirini Theochari, Angeliki Andreopoulou, Spyridoula Vassilouli, Dimitra Giannakopoulou, Eleni Siettou, Eleftheria Tsaltas

**Affiliations:** 1 Psychiatric Hospital of Attica; 2 Athens University Medical School

## Abstract

**Background:**

The relationship of age and symptoms with the performance on non-verbal cognitive planning tasks in schizophrenia could be useful for the development of cognitive remediation programmes.

**Methods:**

During a cross-sectional study, 97 medicated and stabilized patients with chronic schizophrenia (61 males and 36 females, mean age=43.74 years, standard deviation-SD=11.59), which were consecutively referred to our Unit, were assessed using the Stockings of Cambridge (SOC) task of the Cambridge Neuropsychological Test Automated Battery (CANTAB) and the Positive and Negative Syndrome Scale (PANSS). Linear regression analyses were conducted in order to investigate the correlations of symptoms and age with SOC performance.

**Results:**

Age and PANSS total scores negatively correlated with optimal SOC solutions (problems solved in minimum moves) (age: B=-0.05, 95% CI=-0.089, -0–012, df=86, t=-2.599, p=0.011, symptoms: B=-0.047, 95%CI=-0.071, -0.024, df=86, t=-3.982, p<0.001). The effects of total symptoms were driven by positive (B=-0.149, 95%CI=-0.229, -0.068, df=86 t=-3.672, p<0.001), negative (B=-0.087, 95%CI=-0.150, -0.023, df=86, t=-2.717, p=0.008) and general psychopathology symptoms (B=-0.065, 95%CI=-0.108, -0.023, df=86, t=-3.045, p=0.03). PANSS total scores positively correlated with mean excess moves in 2- (B=0.007, 95%CI=0.002, 0.012, df=86, t=2.656, p=0.009), 3- (B=0.014, 95%CI=0.005, 0.023, df=86, t=2.951, p=0.004) and 5-move (B=0.026, 95%CI=0.008, 0.044, df=86, t=2.923, p=0.004) problems and age only in 4- (B=0.026, 95%CI=0.006, 0.046, df=86, t=2.571, p=0.012) and 5-move (B=0.032, 95%CI=0.002, 0.061, df=86, t=2.152, p=0.034) problems. We could not find any association between PANSS scores and age with initial or subsequent thinking times during the SOC task.

**Discussion:**

Cognitive planning deficits in schizophrenia are associated with patients’ symptoms and age. Whereas the effect of symptoms appears to be independent of task difficulty, the age effect emerges when the planning tasks become more complex. The role of drugs remains to be examined in future analyses.

